# HDAC Inhibitors Potentiate Doxorubicin Cytotoxicity in HepG2 Cells and Alter DNA-Repair- and p53-Associated Transcriptional Programs

**DOI:** 10.3390/ijms27209091

**Published:** 2026-10-14

**Authors:** Kaidi Guo, Marcel van Herwijnen, Florian Caiment, Twan van den Beucken

**Affiliations:** Department of Translational Genomics, GROW—Research Institute for Oncology and Reproduction, Maastricht University, 6229 ER Maastricht, The Netherlands

**Keywords:** histone deacetylase inhibitors (HDACis), doxorubicin, HepG2 cells, RNA-seq transcriptomics, DNA repair and p53 signaling, Chemosensitization/drug sensitization

## Abstract

Histone deacetylase inhibitors (HDACis) can enhance responses to DNA-damaging chemotherapy, but how they alter doxorubicin (DOX)-induced transcriptional programs remains incompletely understood. We investigated the effects of valproic acid (VPA) and vorinostat (SAHA) on DOX cytotoxicity and associated molecular responses in HepG2 cells. HDAC inhibition, cell viability, apoptosis, and DOX IC_50_ values were assessed using histone H3 acetylation, cell counting, Alamar Blue assays, and cleaved caspase-3 immunofluorescence. Transcriptomic responses were analyzed by RNA sequencing, differential expression analysis, and Hallmark gene set enrichment analysis, with selected findings evaluated by RT-qPCR, Western blotting, and comparison with published CRISPR/Cas9 DOX-response data. Both HDAC inhibitors increased histone H3 acetylation and enhanced DOX cytotoxicity, markedly reducing DOX IC_50_ values. VPA co-treatment also increased DOX-induced cleaved caspase-3 signaling. RNA-seq showed broad transcriptional remodeling, including depletion of the DNA-repair gene set following VPA treatment and attenuation of canonical p53-responsive genes during HDACi–DOX co-treatment. Several HDACi-repressed DNA-repair genes overlapped with genes whose loss sensitized cells to DOX in a published CRISPR screen. HDAC inhibition therefore enhances DOX cytotoxicity and is accompanied by alterations in DNA-repair-associated and p53-responsive transcriptional programs.

## 1. Introduction

Histone deacetylases (HDACs) regulate protein acetylation by removing acetyl groups from lysine residues on histone and non-histone proteins. Histone deacetylation can promote chromatin compaction and influence transcriptional accessibility, whereas deacetylation of non-histone substrates affects diverse cellular processes, including proliferation, differentiation, cell cycle progression, protein stability, and stress responses [1]. Consequently, dysregulated HDAC activity has been implicated in cancer development and progression, and HDAC inhibitors (HDACis) have emerged as an important class of epigenetic therapeutics. Several HDAC inhibitors have received regulatory approval for hematological malignancies, while their potential in solid tumors continues to be investigated, particularly in combination with other anticancer agents [2,3].

Valproic acid (VPA) and vorinostat (suberoylanilide hydroxamic acid; SAHA) are pharmacologically distinct HDAC inhibitors. VPA is a short-chain fatty acid widely used as an antiepileptic and mood-stabilizing drug that also inhibits predominantly class I and class IIa HDACs. SAHA is a hydroxamic acid HDAC inhibitor with broader activity against zinc-dependent HDACs and is approved for the treatment of cutaneous T-cell lymphomas [2,3]. Both compounds have been extensively studied for their ability to alter cancer cell proliferation, differentiation, and survival, as well as to enhance responses to conventional anticancer therapies.

Doxorubicin (DOX) is an anthracycline chemotherapeutic agent that exerts its anticancer activity predominantly through topoisomerase II inhibition, DNA-damage response, and additional oxidative and cellular stress responses [4,5,6]. Its clinical utility is limited by dose-dependent toxicity, providing a rationale for combination strategies that enhance tumor cell sensitivity to DOX. HDAC inhibition is of particular interest in this context because changes in chromatin accessibility, transcriptional regulation, DNA-damage responses, and apoptotic signaling may modify cellular responses to genotoxic agents [7].

Previous studies have established that HDAC inhibitors can enhance the anticancer effects of DOX in several experimental models. In hepatocellular carcinoma (HCC) cells, Saha et al. showed that VPA and DOX synergistically reduced cell viability in HepG2 and SNU475 cells, with a formal drug-interaction analysis confirming synergy [8]. The combination increased apoptosis, reactive oxygen species generation, autophagy, and intracellular DOX accumulation, suggesting that enhanced DOX internalization contributes to the interaction. Other HDAC inhibitors have also been investigated in combination with DOX. Apicidin enhanced the antitumor effects of DOX in SULF1-expressing liver cancer models [9]. In breast cancer models, Hii et al. demonstrated that several HDAC inhibitors, including givinostat and vorinostat (SAHA), enhanced DOX cytotoxicity in cancer stem cell-enriched and non-stem cell populations [10]. The potentiating effect of SAHA on DOX has also been reported in other tumor types, including triple-negative breast cancer [11] and cervical cancer [12]. Together, these studies establish that HDAC inhibitors can enhance DOX responses through multiple cellular processes, although the nature of these responses varies across compounds and experimental models. VPA was selected based on previous evidence that it enhances DOX cytotoxicity in HepG2 cells, while SAHA was included as a chemically distinct HDAC inhibitor to investigate whether the associated transcriptional responses were shared between the two compounds or were compound-specific. VPA and SAHA are well-established examples of the short-chain fatty acid and hydroxamic acid classes, respectively. Although the ability of HDAC inhibitors to potentiate DOX cytotoxicity has been established, the transcriptional responses accompanying this interaction remain incompletely characterized. In particular, it is unclear to what extent chemically distinct HDAC inhibitors induce shared or compound-specific transcriptional changes and how they modify the transcriptional response to DOX. In the present study, we therefore compared the effects of VPA and SAHA on DOX cytotoxicity and performed transcriptome-wide profiling of HepG2 cells treated with either inhibitor alone or in combination with DOX. HepG2 was selected to build on previous findings demonstrating VPA-mediated potentiation of DOX cytotoxicity in this model [8]. We investigated changes in DNA-repair-associated, metabolic, and p53-responsive transcriptional programs and compared selected HDACi-regulated genes with an independent genome-wide CRISPR/Cas9 screen of DOX sensitivity. Rather than seeking to establish a new drug interaction, our study aimed to characterize the molecular responses accompanying an established sensitizing effect and to identify candidate processes for subsequent functional investigation.

## 2. Results

### 2.1. Validation of HDAC Inhibition and Impact on DOX Cytotoxicity

**Figure 1:**
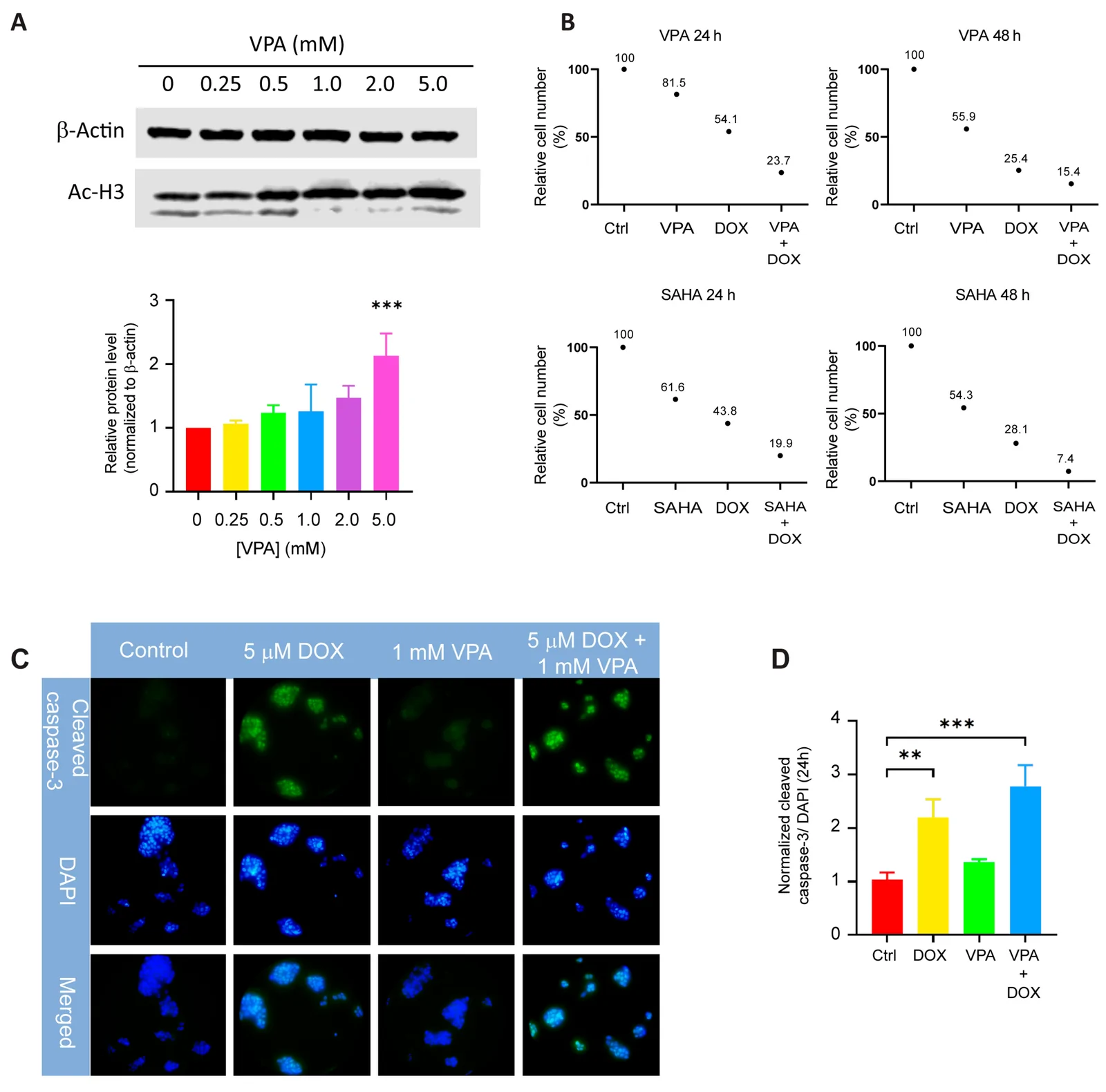
HDAC inhibition enhances DOX-induced cytotoxicity and apoptosis in HepG2 cells. (**A**) Western blot analysis of acetylated histone H3 (Ac-H3) levels in HepG2 cells treated with increasing concentrations of VPA for 24 h. Ac-H3 abundance increased in a concentration-dependent manner, with a marked increase at 5 mM (*** *p* < 0.001), while β-actin served as the loading control. Quantification of Ac-H3 band intensity normalized to β-actin from two independent experiments is shown below the representative blot. (**B**) Effects of VPA (1 mM) or SAHA (1 µM), alone or in combination with DOX (1 µM), on HepG2 cell numbers after 24 and 48 h. Cell counts are expressed relative to the corresponding control. Co-treatment with either of the HDAC inhibitors and DOX reduced cell numbers compared with single-agent treatments. (**C**) Representative immunofluorescence images of cleaved caspase-3 in HepG2 cells treated with VPA and/or DOX for 24 h. Nuclei were counterstained with DAPI. DOX increased cleaved caspase-3 signal, which was further increased following VPA+DOX co-treatment. (**D**) Quantification of cleaved caspase-3 fluorescence intensity normalized to DAPI. Statistical significance was assessed by one-way ANOVA followed by Tukey’s multiple-comparison test (*p* < 0.05, ** *p* < 0.001, *** *p* < 0.0001).

To confirm the HDAC inhibitory activity of valproic acid (VPA), we assessed acetylated histone H3 (Ac-H3) levels in HepG2 cells by Western blot following exposure to increasing VPA concentrations. VPA induced a clear concentration-dependent increase in Ac-H3 abundance, with a pronounced elevation at 5 mM, while β-actin served as the loading control (Figure 1A, *p* < 0.001). The corresponding full Western blot images are provided in Figure S1. We next examined whether HDAC inhibition altered the cytotoxic response to doxorubicin (DOX). HepG2 cells were treated with VPA (1 mM) or another HDAC inhibitor, SAHA (1 µM), either alone or in combination with DOX (1 µM), for 24 and 48 h. Cell counting showed that co-treatment reduced cell numbers compared with the corresponding single-agent treatments, with the effect becoming more pronounced at 48 h (Figure 1B).

To determine whether reduced cell numbers were accompanied by increased apoptotic signaling, cleaved caspase-3 was assessed by immunofluorescence. DOX alone increased the cleaved caspase-3 signal, which was further increased by co-treatment with VPA (Figure 1C). Quantification confirmed a significant increase in cleaved caspase-3 intensity following DOX+VPA co-treatment (*p* < 0.001) relative to control (Figure 1D). Together, these results show that HDAC inhibition enhances DOX-induced cytotoxicity in HepG2 cells and is accompanied by increased apoptotic signaling.

### 2.2. HDAC Inhibitors Sensitize HepG2 Cells to DOX by Reducing the DOX IC_50_

**Figure 2:**
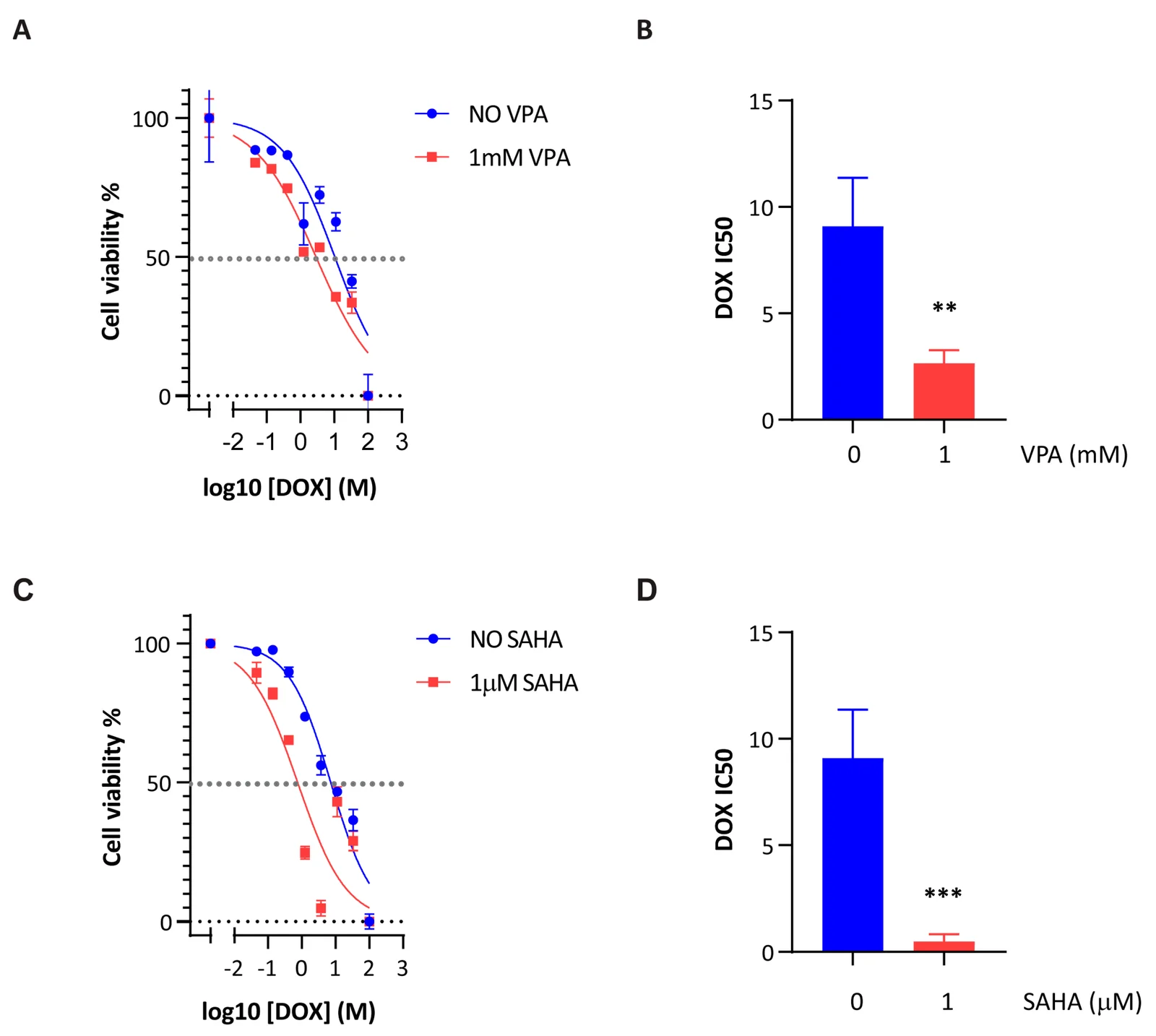
HDAC inhibitors reduce the DOX IC_50_ in HepG2 cells. (**A**) Representative DOX dose–response curves in the presence or absence of 1 mM VPA. (**B**) Mean DOX IC_50_ values from three independent biological experiments. Co-treatment with VPA significantly reduced the DOX IC_50_ (** *p* < 0.01). (**C**) Representative DOX dose–response curves in the presence or absence of 1 μM SAHA. (**D**) Mean DOX IC_50_ values from three independent biological experiments. Co-treatment with SAHA significantly reduced the DOX IC_50_ (*** *p* < 0.001). Data are presented as mean ± SD. IC_50_ values were calculated by nonlinear regression analysis using GraphPad Prism version 8.4.3. Cell viability was assessed using the Alamar Blue assay after 24 h of drug exposure. Each condition was measured in technical triplicate across three independent biological experiments.

To quantify the effect of HDAC inhibition on DOX sensitivity, we determined DOX half-maximal inhibitory concentration (IC_50_) values in the presence or absence of VPA or SAHA. Co-treatment with 1 mM VPA shifted the DOX dose–response curve leftward (Figure 2A), resulting in a significant reduction in the calculated IC_50_ from 9.1 µM to 2.7 µM across three independent experiments (*p* < 0.01) (Figure 2B). Similarly, co-treatments with 1 µM SAHA significantly reduced the DOX IC_50_ from 9.1 µM to 0.5 µM (*p* < 0.001) (Figure 2C,D). These data demonstrate that both VPA and SAHA increase the sensitivity of HepG2 cells to DOX.

### 2.3. HDAC Inhibition Remodels the HepG2 Transcriptome and Alters Stress-Associated Programs

**Figure 3:**
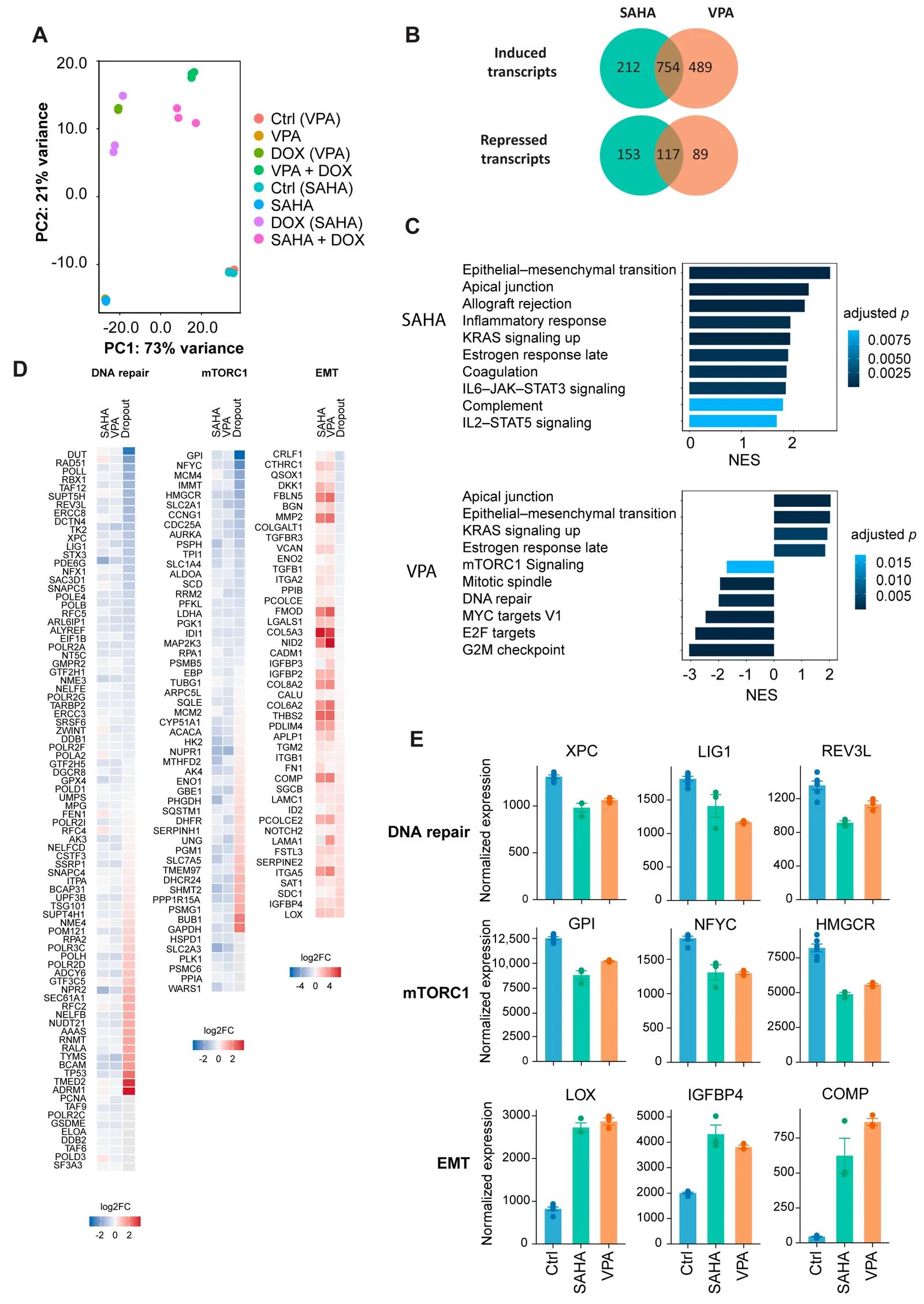
HDAC inhibition remodels transcriptional programs associated with DNA repair, metabolism, and phenotypic remodeling. (**A**) Principal component analysis (PCA) of RNA-seq profiles from HepG2 cells treated for 48 h with control medium, VPA, SAHA, DOX, VPA+DOX or SAHA+DOX. PC1 (73%) separates DOX-exposed from non-DOX conditions, while PC2 (21%) captures variance predominantly associated with HDAC inhibition. (**B**) Venn diagrams showing overlap of differentially expressed genes (adjusted *p* < 0.05) upregulated (**top**) or downregulated (**bottom**) by SAHA and VPA relative to control. (**C**) Hallmark gene set enrichment analysis (GSEA) for SAHA-treated (**top**) and VPA-treated (**bottom**) cells, depicting normalized enrichment scores (NESs) and adjusted *p*-values. (**D**) Heatmaps showing log2 fold-changes in genes within the Hallmark DNA-repair, mTORC1 signaling, epithelial–mesenchymal transition gene sets. Genes are additionally annotated with DOX-response scores from a published CRISPR/Cas9 loss-of-function screen. Negative dropout scores indicate increased DOX sensitivity following gene knockout, whereas positive scores indicate enrichment of knockout cells during DOX exposure, consistent with increased resistance. (**E**) DESeq2-normalized RNA-seq expression levels of representative genes from the selected pathways, including DNA-repair-associated genes (*XPC*, *LIG1*, *REV3L*), mTORC1/metabolic genes (*GPI*, *NFYC*, *HMGCR*), and EMT/ECM-associated genes (*LOX*, *IGFBP4*, *COMP*).

To characterize transcriptional responses associated with HDACi-mediated DOX sensitization, we performed transcriptome profiling of HepG2 cells treated with VPA or SAHA in the presence or absence of DOX for 48 h. RNA sequencing was performed using three independent biological replicates per condition. Principal component analysis (PCA) showed that DOX exposure was the major driver of transcriptional variance, with PC1 explaining 73% of total variance and separating DOX-exposed from non-DOX samples (Figure 3A). PC2 accounted for 21% of the variance and largely reflected differences associated with HDAC inhibitor treatment. Biological replicates clustered tightly, indicating high reproducibility.

Differential expression analysis (adjusted *p* < 0.05) revealed both shared and inhibitor-specific transcriptional responses (Figure 3B). Among HDACi-induced transcripts, 754 genes were commonly upregulated by SAHA and VPA, whereas 212 and 489 genes were uniquely induced by SAHA or VPA, respectively. A similar pattern was observed for downregulated genes, with 117 transcripts commonly downregulated and additional subsets uniquely repressed by SAHA (153 genes) or VPA (89 genes). Overall, SAHA and VPA produced broad and partially overlapping transcriptional responses, with a greater number of genes induced than repressed.

Hallmark gene set enrichment analysis (GSEA) identified several shared transcriptional responses to VPA and SAHA treatment (Figure 3C). Enriched pathways included apical junction and epithelial–mesenchymal transition (EMT), whereas depleted pathways included mTORC1 signaling. Notably, the Hallmark DNA-repair gene set was significantly depleted following VPA treatment (normalized enrichment score (NES) = –2.00, adjusted *p* = 5.5 × 10^–5^), while SAHA showed a similar but non-significant trend (NES = –1.35, adjusted *p* = 0.09). These findings indicate that HDAC inhibition, particularly by VPA, is associated with reduced expression of DNA-repair-associated transcriptional programs.

To assess whether HDACi-regulated genes overlap with genes previously implicated in DOX sensitivity, we compared our transcriptomic data with a published genome-wide CRISPR/Cas9 loss-of-function screen performed in human RPE1-hTERT (*TP53*−/−, Cas9) cells exposed to DOX [13] (Figure 3D). The corresponding full gene set heatmaps are provided in Figure S2. In this dataset, negative dropout scores indicate increased DOX sensitivity following gene knockout. Several DNA-repair and damage-tolerance genes repressed by HDAC inhibition were also among genes whose knockout sensitized cells to DOX, including *REV3L* (dropout −1.41), *LIG1* (dropout −1.08), and *XPC* (dropout −1.11). This overlap identifies candidate genes that may link HDACi-associated transcriptional changes to DOX sensitivity, although the CRISPR screen was performed in a different cellular and experimental context.

Beyond DNA-repair-associated genes, comparison with the CRISPR dataset identified additional HDACi-regulated genes previously implicated in the response to DOX. Within the mTORC1/metabolic signature, HDACi repressed several genes whose knockout sensitized cells to DOX, including *GPI* (dropout −2.88), *NFYC* (dropout −2.07), *IMMT* (dropout −1.48), and *HMGCR* (dropout −1.35). HDACi also induced an EMT/ECM remodeling program. Among these genes, the CRISPR dataset contained genes whose knockout increased DOX sensitivity (e.g., *DKK1*, *CRLF1*, *FBLN5*, *QSOX1*) and genes whose knockout increased DOX resistance (e.g., *LOX*, *IGFBP4*, *SDC1*, *SAT1*, *LAMC1*, *COMP*). Thus, HDACi-regulated EMT/ECM genes showed heterogeneous relationships with DOX response in the external CRISPR dataset. The normalized RNA-seq expression levels of selected genes from these pathways are shown in Figure 3E, illustrating the transcriptional changes associated with VPA and SAHA treatment. Collectively, these analyses show that HDAC inhibition alters transcriptional programs associated with DNA repair, metabolism, and phenotypic remodeling, several components of which overlap with genes implicated in DOX response in an independent genetic screen.

### 2.4. HDAC Inhibition Modifies the DOX-Induced Transcriptional Response

**Figure 4:**
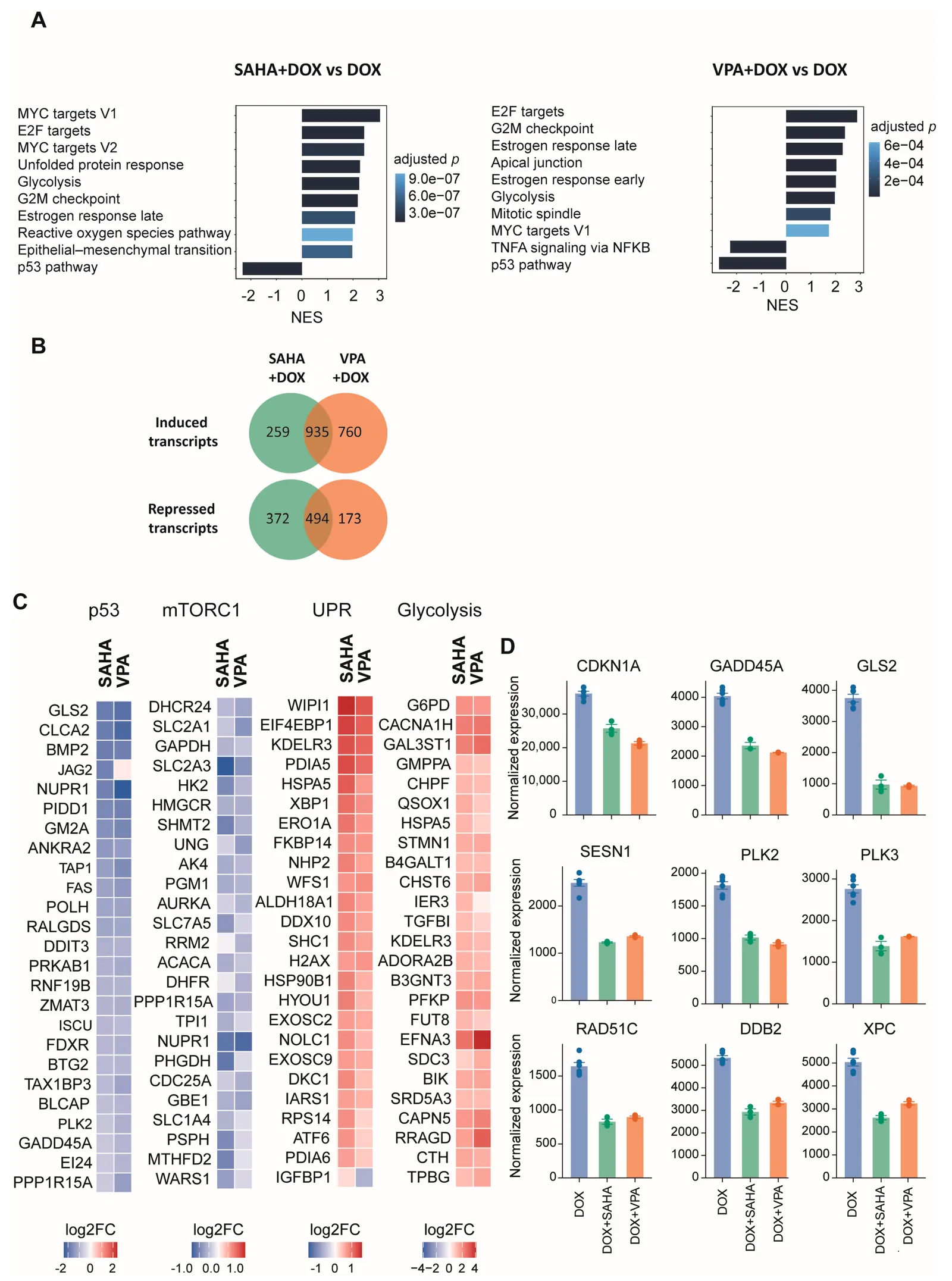
HDAC inhibition modifies the DOX-induced transcriptional response. (**A**) Hallmark gene set enrichment analysis (GSEA) comparing SAHA+DOX versus DOX (**left**) and VPA+DOX vs. DOX (**right**) after 48 h, showing normalized enrichment scores (NESs) for the most significantly altered Hallmark gene sets. (**B**) Venn diagrams showing overlap of significantly upregulated (**top**) and downregulated (**bottom**) transcripts (adjusted *p* < 0.05) in SAHA+DOX versus DOX and VPA+DOX vs. DOX contrasts. (**C**) Heatmaps showing gene-level log2 fold-change patterns within selected Hallmark gene sets, including p53 pathway, mTORC1 signaling, unfolded protein response (UPR), and glycolysis, for SAHA+DOX vs. DOX and VPA+DOX versus DOX. (**D**) DESeq2-normalized RNA-seq expression levels of selected p53-associated genes (*CDKN1A*, *GADD45A*, *GLS2*, *SESN1*, *PLK2*, *PLK3*) and DNA-damage-response genes (*RAD51C*, *DDB2*, *XPC*) in DOX-, DOX+SAHA-, and DOX+VPA-treated HepG2 cells.

Next, we investigated whether HDAC inhibition modifies the transcriptional response to DOX by comparing VPA+DOX and SAHA+DOX with DOX alone after 48 h (Figure 4). Hallmark GSEA revealed substantial pathway-level differences in both combination treatments (Figure 4A), including modulation of p53-associated stress responses, growth-related programs, and metabolic pathways. Several signatures were affected by both HDAC inhibitors, although the magnitude and statistical significance of these effects differed between VPA and SAHA, indicating both shared and inhibitor-specific responses. Notably, mTORC1 signaling was significantly altered following SAHA+DOX treatment and showed a similar but non-significant trend following VPA+DOX treatment (NES = 1.29, adjusted *p* = 0.08).

Differential expression analysis further revealed a substantial shared set of HDACi-dependent transcripts regulated during DOX exposure, alongside distinct inhibitor-specific gene sets (Figure 4B). Heatmaps of representative Hallmark programs showed coordinated gene-level changes across key pathways (Figure 4C). The corresponding full gene set heatmaps are provided in Figure S3. Multiple p53 pathway genes, including *CDKN1A*, *GADD45A*, *GLS2*, *SESN1*, *PLK2*, and *PLK3*, as well as DNA-damage-response genes including *DDB2*, *XPC*, and *RAD51C*, showed reduced induction in HDACi + DOX-treated cells compared with that in DOX alone (Figure 4D). Together, these results demonstrate that HDAC inhibition substantially modifies the DOX-induced transcriptional response, including attenuation of several p53-associated and DNA-damage-response genes.

### 2.5. HDAC Inhibitors Attenuate DOX-Induced p53 Transcriptional Outputs and Reduce p53 Accumulation

**Figure 5:**
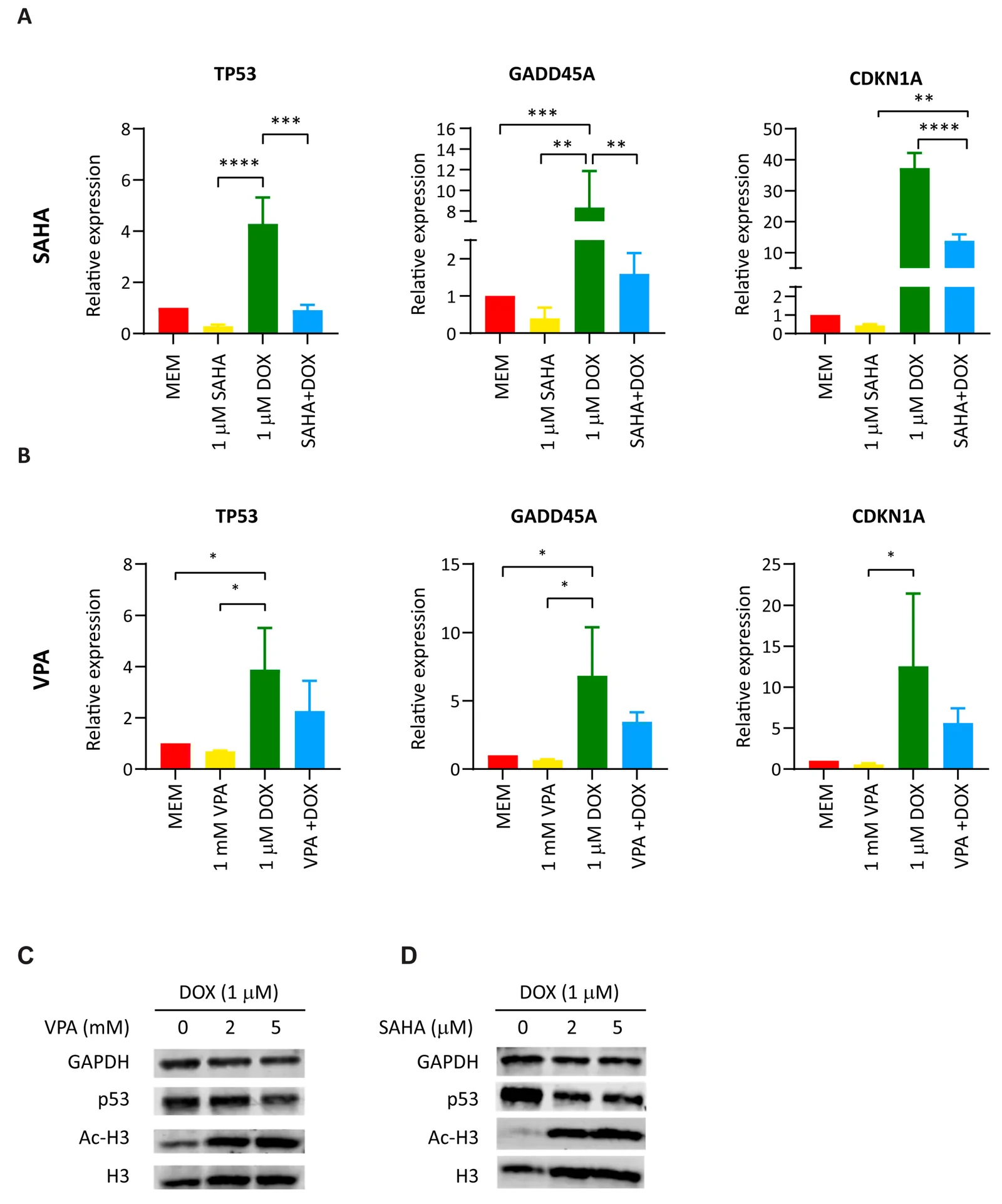
HDAC inhibitors attenuate DOX-induced p53-associated transcriptional responses and p53 accumulation. (**A**,**B**) RT–qPCR analysis of TP53 and the p53-responsive genes *GADD45A* and *CDKN1A* in HepG2 cells treated for 48 h with DOX (1 µM) alone or in combination with SAHA (1 µM) (**A**) or VPA (1 mM) (**B**). Expression was normalized to *HPRT* and is shown relative to control (mean ± SD) from three independent biological experiments. Statistical significance was assessed by one-way ANOVA followed by Tukey’s multiple comparisons test (* *p* < 0.05, ** *p* < 0.01, *** *p* < 0.001, **** *p* < 0.0001). (**C**,**D**) Western blot analysis of p53 protein abundance in HepG2 cells treated with DOX (1 µM) for 24 h in the presence of increasing concentrations of VPA (0, 2, and 5 mM; (**C**)) or SAHA (0, 2, and 5 µM; (**D**)). GAPDH served as the loading control for p53, while Ac-H3 and total histone H3 were assessed to demonstrate increased histone acetylation following HDAC inhibitor treatment.

To validate the RNA-seq observation that HDAC inhibition alters p53-associated transcriptional outputs during DOX exposure, we quantified selected p53-responsive genes by reverse transcription quantitative PCR (RT-qPCR). HepG2 cells were treated with VPA or SAHA ± DOX for 48 h, and transcript levels of *CDKN1A* and *GADD45A* were assessed as markers of the canonical p53-associated DNA-damage response. DOX alone markedly induced both *CDKN1A* and *GADD45A*, consistent with activation of a classical DNA-damage response. In contrast, co-treatment with DOX and either of the HDAC inhibitors significantly attenuated induction of both genes, with the strongest attenuation observed for *GADD45A*. The effect was more pronounced following SAHA treatment, which reduced DOX-induced expression of *GADD45A* and *CDKN1A* and also decreased *TP53* mRNA levels (Figure 5A,B).

We then examined p53 protein abundance by Western blot. DOX exposure increased p53 protein levels, whereas co-treatment with VPA or SAHA reduced DOX-induced p53 accumulation in a concentration-dependent manner (Figure 5C,D). Both inhibitors increased Ac-H3 levels, confirming HDAC inhibition under the tested treatment conditions. The corresponding full Western blot images are provided in Figure S1. Thus, enhanced DOX cytotoxicity following HDAC inhibition occurred despite attenuation of canonical p53-associated transcriptional responses and reduced DOX-induced p53 accumulation.

## 3. Discussion

In this study, we investigated how histone deacetylase inhibition modifies doxorubicin (DOX) cytotoxicity and associated transcriptional responses in HepG2 cells. Using two pharmacologically distinct HDAC inhibitors, valproic acid (VPA) and SAHA, we show that HDAC inhibition increases the sensitivity of HepG2 cells to DOX, as evidenced by reduced cell numbers, increased cleaved caspase-3 signal, and pronounced reductions in the DOX IC_50_. These findings are consistent with previous reports describing potentiating effects of HDAC inhibitors on DOX activity in diverse cancer models [11,14,15]. Transcriptome profiling further showed that HDAC inhibition induces broad transcriptional changes affecting several cellular programs associated with DNA-damage responses, metabolism, stress adaptation, and phenotypic remodeling.

The principal contribution of the present study is the comparative characterization of transcriptional responses accompanying HDACi-mediated DOX sensitization rather than the demonstration of the sensitizing interaction itself. Our findings identify candidate processes that can be investigated experimentally. In particular, the overlap between HDACi-repressed DNA-repair-associated genes and genes implicated in DOX sensitivity by an independent CRISPR screen suggests that the contributions of *REV3L*, *LIG1*, and *XPC* could be examined through targeted perturbation in HepG2 cells. Similarly, the attenuation of canonical p53-associated transcription during co-treatment raises the question of whether this response contributes to increased DOX sensitivity or reflects a secondary consequence of treatment-induced cellular stress. Earlier transcriptional measurements and targeted functional studies would help distinguish these possibilities.

A prominent finding of the RNA-seq analysis was the modulation of DNA-repair-associated transcriptional programs. Hallmark GSEA revealed significant depletion of the DNA-repair gene set following VPA treatment, whereas SAHA showed a similar but non-significant trend. To explore whether genes within these transcriptional responses have previously been functionally linked to DOX sensitivity, we compared the HDACi-regulated genes with results from a published genome-wide CRISPR/Cas9 loss-of-function screen. Several HDACi-repressed DNA-repair and damage-tolerance genes (e.g., *REV3L*, *LIG1*, *XPC*) coincide with genetic perturbations that sensitize cells to DOX [13]. Thus, the RNA-seq and CRISPR datasets provide complementary but distinct information: HDAC inhibition reduces expression of these genes in HepG2 cells, whereas their genetic loss was associated with increased DOX sensitivity in an independent cellular model. This overlap identifies DNA-repair- and damage-tolerance-associated genes as candidates for future functional investigation in the context of HDACi-mediated DOX sensitization. However, because DNA-repair activity was not directly measured in the present study, these transcriptional changes should not be interpreted as evidence of impaired DNA-repair capacity.

Previous studies provide biological precedents for such a relationship. Adimoolam et al. demonstrated that HDAC inhibition can transcriptionally repress homologous recombination genes, including *RAD51* and *BRCA1/2*, resulting in impaired RAD51 focus formation and defective DNA repair [16]. Robert et al. showed that HDAC inhibition can affect DNA double-strand break processing by altering CtIP/Sae2 and Exo1 stability [17]. These studies demonstrate that HDAC inhibition can interfere with DNA-repair processes in other experimental systems and therefore support the biological plausibility of the transcriptional changes observed here. Whether similar functional impairment occurs in HepG2 cells under the present treatment conditions remains to be established.

The comparison with the published CRISPR screen should likewise be interpreted as supportive rather than as direct functional validation. The screen was performed in RPE1-hTERT TP53−/− cells under experimental conditions that differ from those used here, and complete gene knockout is not equivalent to the partial transcriptional changes induced by HDAC inhibition. Nevertheless, the overlap identifies genes and pathways that may be relevant to DOX response and provides a useful framework for prioritizing candidate mechanisms for future investigation.

HDAC inhibition also altered transcriptional programs related to metabolism and mTORC1 signaling. Several HDACi-repressed genes within these programs, including *GPI*, *NFYC*, *IMMT*, and *HMGCR*, were associated with altered DOX sensitivity in the published CRISPR screen. Because mTORC1 signaling contributes to cellular metabolism, growth, and adaptation to stress [18,19], modulation of these pathways may influence cellular responses to DOX. However, metabolic activity and mTORC1 signaling were not directly assessed in the present study, and their contribution to HDACi-mediated DOX sensitization therefore remains to be determined.

HDAC inhibition was additionally associated with enrichment of epithelial–mesenchymal transition (EMT) and extracellular matrix (ECM) transcriptional signatures. EMT-associated programs have frequently been linked to altered therapeutic responses [20]. In the external CRISPR dataset, however, HDACi-regulated EMT/ECM genes were associated with both increased DOX sensitivity and increased DOX resistance upon knockout. Thus, rather than indicating a uniform shift toward either sensitivity or resistance, the observed signature most likely reflects broader transcriptional and phenotypic remodeling. The functional relevance of these changes to DOX response remains unclear.

A notable finding was the attenuation of canonical p53-associated transcriptional responses during HDACi-DOX co-treatments. DOX alone induced *CDKN1A* and *GADD45A*, whereas co-treatment with HDAC inhibitors reduced their induction. Consistent with these transcriptional findings, Western blot analysis revealed reduced DOX-induced p53 accumulation under increasing HDACi doses, despite robust histone H3 hyperacetylation confirming effective HDAC inhibition. *CDKN1A* and *GADD45A* are involved in cellular responses to DNA damage, including cell cycle regulation and stress adaptation. Their attenuated induction raises the possibility that HDACi–DOX co-treatment alters the balance between damage-responsive growth arrest and cell death. However, because transcriptional responses were measured after 48 h of treatment, when changes in cell number were already evident, reduced expression of these genes may also reflect a secondary response to treatment-induced cellular stress rather than a mechanism contributing to sensitization. Thus, increased cytotoxicity during HDACi–DOX co-treatment was observed despite attenuation of canonical p53-associated transcriptional responses.

The relationship between HDAC inhibition and p53 signaling is complex because HDAC inhibitors affect both histone and non-histone protein acetylation. p53 acetylation can influence protein stability, DNA binding, and transcriptional selectivity [21,22,23,24,25]. However, p53 acetylation was not measured in the present study, and altered post-translational regulation of p53 therefore remains one possible explanation rather than a demonstrated mechanism. We did not assess p53 acetylation, MDM2-dependent regulation, or proteasomal turnover, and therefore cannot determine whether the reduced p53 accumulation reflects altered transcription, protein stability, or degradation. Importantly, previous studies have shown that HDAC inhibitor-induced cytotoxicity can occur through both p53-dependent and p53-independent mechanisms [26,27]. Vorinostat has also been reported to potentiate DOX cytotoxicity in several tumor cell lines, with the relationship between HDAC inhibition, p53 acetylation, and drug response varying between cellular contexts [12]. Because p53 regulates both growth-arrest and pro-apoptotic responses, attenuation of selected p53-responsive genes does not establish that the enhanced cytotoxicity observed here occurs through a p53-independent mechanism. Together, these observations are consistent with a context-dependent relationship between HDAC inhibition and p53 signaling.

Several limitations should be considered. First, the present study was performed in a single hepatic cancer-derived cell line. HepG2 cells should not be considered representative of the biological heterogeneity of adult hepatocellular carcinoma. Validation in additional authenticated HCC cell lines with different TP53 backgrounds, as well as in vivo studies, would be required to determine the generalizability of the observed responses. Second, RNA-seq was performed after 48 h of treatment, when changes in cell number were already evident. The observed transcriptional responses may include secondary consequences of treatment-induced cellular stress. The present data therefore cannot establish whether the altered transcriptional programs contribute to DOX sensitization or arise as a consequence of the enhanced treatment response. Furthermore, although RNA-seq and comparison with published CRISPR screening data identified DNA-repair-associated genes and pathways of potential relevance to DOX sensitivity, DNA-damage accumulation and DNA-repair activity were not directly assessed. Direct functional DNA-damage and -repair assays, together with time-resolved analyses and genetic perturbation studies, would be required to determine whether the observed transcriptional changes contribute to DOX sensitization. Third, although HDAC inhibition altered DOX-induced p53 accumulation and p53-associated transcription, the underlying molecular basis of this effect was not investigated. Finally, cleaved caspase-3 was assessed only for VPA–DOX co-treatment and under different DOX exposure conditions from those used for transcriptomic profiling, while p53 protein abundance was measured at an earlier time point and at higher HDAC inhibitor concentrations. These differences limit the direct mechanistic integration of the apoptosis, transcriptional, and protein-level findings.

Overall, our findings demonstrate that HDAC inhibition increases DOX sensitivity in HepG2 cells and substantially modifies the transcriptional response to DOX. These changes include attenuation of DNA-repair-associated and canonical p53-responsive transcriptional programs, together with broader alterations in metabolic and phenotypic remodeling pathways. Rather than establishing a single mechanism of HDACi-mediated DOX sensitization, the present data identify several candidate processes that may contribute to the enhanced cytotoxic response. Further functional studies will be required to determine their causal contributions and to establish how these responses vary across different HCC models.

## 4. Materials and Methods

### 4.1. Cell Culture

The liver cancer cell line HepG2 was purchased from the American Type Culture Collection (ATCC, Manassas, VA, USA; HB-8065; RRID:CVCL_0027). HepG2 cells were cultured in Minimum Essential Medium (MEM; Gibco, Cat. #11095-080, Grand Island, NY, USA) supplemented with 10% fetal bovine serum (FBS; Gibco, Cat. #10099-141, Grand Island, NY, USA) and 100 U/mL penicillin–streptomycin (Gibco, Cat. #15140-122, Grand Island, NY, USA) in a humidified incubator with 5% CO_2_ at 37 °C. Unless otherwise specified, the culture medium was replaced every 2 days.

### 4.2. RNA Extraction and RT-qPCR

HepG2 cells were seeded in 6-well plates at a density of 2 × 10^5^ cells per well and treated with two different HDAC inhibitors, 1 mM VPA (Sigma-Aldrich, St. Louis, MO, USA; Cat. #P4543) or 1 μM SAHA (Sigma-Aldrich, St. Louis, MO, USA; Cat. #SML0061)) in the presence or absence of 1 μM DOX (Sigma-Aldrich, St. Louis, MO, USA; Cat. #D1515), for 24 or 48 h. RNA-seq samples were prepared from the 48 h groups only under the same treatment conditions. After treatment, cells were detached by trypsin and resuspended. An aliquot was taken to assess cell numbers using an automated cell Countess II Automated Cell Counter (Thermo Fisher Scientific, Waltham, MA, USA). The remaining cells were pelleted, washed once with PBS, and then lysed in 0.5 mL TRIzol™ reagent (Thermo Fisher Scientific, Cat# 15596026, Waltham, MA, USA). Lysates were incubated at RT for 5 min to fully dissociate nucleoprotein complexes. Subsequently, 0.2 mL of chloroform (Thermo Fisher Scientific, Cat# C298-500) was added per 1 mL of TRIzol reagent. The samples were vigorously shaken and centrifuged at 12,000× *g* for 15 min at 4 °C to separate the phases. The mixture was separated into the lower red phenol–chloroform, the intermediate phase, and the colorless upper aqueous phase. The aqueous phase was carefully transferred to a new RNase-free tube, and 0.5 mL of isopropanol (Thermo Fisher Scientific, Cat# A415-4) was added per 1 mL of TRIzol™ reagent to precipitate RNA. Samples were incubated at RT for 10 min and centrifuged at 12,000× *g* for 10 min at 4 °C. The RNA pellet was washed twice with 75% ethanol, briefly air-dried, and resuspended in 20 μL of nuclease-free water (Thermo Fisher Scientific, Cat# AM9932). RNA was resuspended by incubation at 55 °C for 15 min.

Reverse transcription was performed using the iScript™ cDNA Synthesis Kit (Bio-Rad, Hercules, CA, USA). Subsequent gene expression analysis by RT-qPCR was performed using a CFX96 Real-Time PCR Detection System (Bio-Rad) in a total reaction volume of 10 μL. Each reaction contained 5 μL iQ™ SYBR Green Supermix (2×; Bio-Rad), 3 μL primer mix containing forward and reverse primers at 3 μM each (final concentration 0.9 μM each), and 2 μL diluted cDNA. Amplification was performed at 95 °C for 3 min, followed by 40 cycles of 95 °C for 10 s and 55 °C for 30 s. Gene expression was normalized to *HPRT*, and relative expression was calculated using the 2^−ΔΔCt^ method. The primer sequences (5′–3′) were as follows: *HPRT*, forward CATTATGCTGAGGATTTGGAAAGG and reverse CTTGAGCACACAGAGGGCTACA; *TP53*, forward GGCCCACTTCACCGTACTAA and reverse GTGGTTTCAAGGCCAGATGT; *CDKN1A*, forward GACACCACTGGAGGGTGACT and reverse CAGGTCCACATGGTCTTCCT; and *GADD45A*, forward CCCCACCTTATCCATCCCTT and reverse CGATCCTGCAGTTTGCAATATTGA. The target genes analyzed included *TP53*, a tumor suppressor that regulates cell cycle arrest and apoptosis; *CDKN1A* (p21), a cyclin-dependent kinase inhibitor that mediates p53-dependent growth arrest; and *GADD45A*, a stress-responsive gene involved in DNA repair, cell cycle control, and apoptosis.

### 4.3. Cell Viability Assay by Alamar Blue

To evaluate the impact of HDAC inhibitors on the cytotoxicity of DOX in HepG2 cells, cell viability was assessed using Alamar Blue reagent (Thermo Fisher Scientific, Cat#DAL1100). HepG2 cells were seeded in 96-well plates at a density of 5000 cells per well in 100 μL of complete culture medium and incubated overnight at 37 °C with 5% CO_2_. The next day, cells were treated according to the following groups: DOX (±VPA): DOX starting at 100 μM, followed by 3-fold serial dilutions, either alone or in combination with 1 mM VPA. DOX (±SAHA): DOX 3-fold dilutions starting from 100 μM, with or without 1 μM SAHA. Following 24 h of treatment with drugs, 10% (*v*/*v*) Alamar Blue reagent was added directly to each well, and cells were incubated for an additional 4 h. Fluorescence intensity was measured using a CLARIOstar^®^ microplate reader (BMG LABTECH, Ortenberg, Germany) (excitation: 560 nm, emission: 590 nm). Cell viability was expressed as a percentage of untreated controls, and IC_50_ values were calculated by nonlinear regression analysis using GraphPad Prism version 8.4.3 software. Each treatment condition was tested in three independent biological replicates.

### 4.4. Immunofluorescence Staining

HepG2 cells were seeded on sterile glass coverslips in 6-well plates at 2 × 10^5^ cells per well and cultured overnight. Cells were then treated with 5 µM doxorubicin (DOX; Sigma-Aldrich, D1515, St. Louis, MO, USA), 1 mM valproic acid (VPA; Sigma-Aldrich, P4543), or their combination for 24 h. After treatment, cells were fixed with 4% paraformaldehyde (Sigma-Aldrich, P6148) for 20 min at room temperature (RT), washed with PBS, and blocked in 1% bovine serum albumin (BSA; Sigma-Aldrich, A9647) for 30 min. Cells were incubated overnight at 4 °C with cleaved caspase-3 primary antibody (1:500, Cell Signaling Technology, 9664, Danvers, MA, USA), followed by Alexa Fluor 488-conjugated secondary antibody (1:1000, Thermo Fisher Scientific, A-11008) for 1 h at RT in the dark. Nuclei were counterstained with DAPI (1 µg/mL, Sigma-Aldrich, D9542) for 30 min. Coverslips were mounted using VECTASHIELD^®^ Antifade Mounting Medium (Vector Laboratories, Cat# H-1000-10, Newark, CA, USA) to minimize fluorescence quenching, and images were acquired by fluorescence microscopy (Nikon Corporation, Tokyo, Japan) to assess caspase-3 activation and nuclear morphology. For each well, three non-overlapping fields of view were imaged for both DAPI and cleaved caspase-3 channels; fluorescence intensities were quantified in ImageJ version 1.51n (National Institutes of Health, Bethesda, MD, USA); and the relative cleaved caspase-3/ DAPI fluorescence intensity was calculated.

### 4.5. Western Blot Analysis

HepG2 cells were seeded in 6-well plates at a density of 5 × 10^5^ cells per well and cultured overnight at 37 °C with 5% CO_2_. Cells were treated with different concentrations of VPA or SAHA, either alone or in combination with DOX, for 24 h according to the following groups: VPA groups: medium only, 2 mM VPA, 5 mM VPA; 1 μM DOX, 1 μM DOX + 2 mM VPA, 1 μM DOX + 5 mM VPA. SAHA groups: medium only, 2 μM SAHA, 5 μM SAHA; 1 μM DOX, 1 μM DOX + 2 μM SAHA, 1 μM DOX + 5 μM SAHA. Following treatment, cells were washed with ice-cold PBS and lysed using RIPA buffer supplemented with a protease and phosphatase inhibitor cocktail (Thermo Fisher Scientific, Cat# A32961). A volume of 300 μL of lysis buffer was added per well. Cell lysates were collected using a cell scraper (Corning, Cat# 3010, Corning, NY, USA), incubated on ice for 30 min, and centrifuged at 5000× *g* for 10 min at 4 °C. Supernatants were collected for protein quantification using a BCA protein assay kit (Thermo Fisher Scientific, Cat# 23227). Equal amounts of protein were mixed with 6× SDS loading buffer, denatured at 95 °C for 5 min, and separated by SDS-PAGE using NuPAGE™ 10% Bis-Tris Mini Gels (Thermo Fisher Scientific, Cat# NP0302BOX). Proteins were transferred using the Trans-Blot Turbo RTA Mini 0.2 µm Nitrocellulose Transfer Kit (Bio-Rad, Cat. #1704270) and the Trans-Blot Turbo Transfer System (Bio-Rad). Membranes were blocked with Intercept™ (TBS) Blocking Buffer (LI-COR Biosciences, Cat# 927-60001, Lincoln, NE, USA) for 1 h at room temperature, followed by overnight incubation at 4 °C with primary antibodies, all diluted 1:1000 in blocking buffer: GAPDH (mouse monoclonal, Cell Signaling Technology, Cat# 97166), anti-β-actin (MP Biomedicals, Santa Ana, CA, USA), p53 (rabbit monoclonal, Cell Signaling Technology, Cat# 2524), acetyl-histone H3 (Lys9) (rabbit polyclonal, Cell Signaling Technology, Cat# 9649), total histone H3 (rabbit polyclonal, Cell Signaling Technology, Cat# 9715). After washing three times with TBST (TBS + 0.1% Tween-20), membranes were incubated with IRDye^®^ secondary antibodies (LI-COR Biosciences, Lincoln, NE, USA) diluted 1:10,000, goat anti-mouse IRDye 680RD (Cat# 926-68070) or goat anti-rabbit IRDye 800CW (Cat# 926-32211). Membranes were imaged using the Odyssey^®^ CLx Imaging System (LI-COR Biosciences). Band intensities were quantified using ImageJ software version 1.51n and normalized to β-actin, GAPDH or total histone H3 as indicated.

### 4.6. Poly-A RNA Sequencing

Poly(A) RNA libraries were generated using the Zephyr G3^®^ NGS automated system (PerkinElmer, Shelton, CT, USA) with the NEXTFLEX^®^ Rapid Directional RNA-Seq Kit 2.0 (NOVA-5198-02, PerkinElmer, Shelton, CT, USA). Library preparation included NEXTFLEX^®^ Poly(A) Beads 2.0 (NOVA-512992, PerkinElmer) for mRNA enrichment and NEXTFLEX^®^ Unique Dual Index Barcodes (NOVA-512923, PerkinElmer) for sample multiplexing. Each library was prepared using 100 ng of total RNA as input, with RNA integrity numbers (RINs) ≥8. A total of 13 PCR cycles were performed during library amplification. Sequencing was performed on the Illumina NovaSeq 6000 (Illumina, San Diego, CA, USA) using an S2 flow cell, generating paired-end reads with 100 cycles per read.

### 4.7. Bioinformatics Analysis

RNA-seq data were processed using the R-ODAF pipeline [28]. The human GRCh38 reference genome was used for read alignment with STAR (version 2.7.1), and gene expression was quantified using RSEM (version 1.3.3) [29]. Differential expression analysis was performed using DESeq2 (version 1.40.2) [30], with an adjusted *p* value < 0.05 considered statistically significant. Heatmaps were generated using the pheatmap package (version 1.0.13) in R (version 4.3.1). Principal component analysis (PCA) was performed using DESeq2 (version 1.40.2) and visualized with ggplot2 (version 3.5.1) [31]. Gene set enrichment analysis (GSEA) was conducted using fgsea (version 1.26.0) with MSigDB Hallmark gene sets. Genes were ranked according to the DESeq2 Wald test statistic. Statistical significance of gene set enrichment was assessed using Benjamini–Hochberg false discovery rate (FDR) correction, with adjusted *p* values < 0.01 considered significant. Expression of selected genes was visualized using the tidyplots package (version 0.4.0) in R [32].

### 4.8. Statistical Analysis

Unless otherwise indicated, data are presented as mean ± standard deviation (SD) from at least three independent biological experiments. Statistical analyses were performed using GraphPad Prism version 8.4.3. Comparisons between two groups were performed using unpaired Student’s *t*-test, while one-way or two-way ANOVA followed by appropriate post hoc tests (Tukey’s or Bonferroni) were used for multiple-group comparisons. Differences were considered statistically significant at *p* < 0.05. Graphs for non-transcriptomic experimental data were generated using GraphPad Prism version 8.4.3; transcriptomic visualizations were generated using the indicated R packages.

## Data Availability

The datasets generated during the current study are available under the ArrayExpress accession E-MTAB-16713.

## Supplementary Materials

The following supporting information can be downloaded at https://www.mdpi.com/article/10.3390/ijms27209091/s1.

## References

[1] Banerjee S., Adhikari N., Amin S.A., Jha T. (2019). Histone deacetylase 8 (HDAC8) and its inhibitors with selectivity to other isoforms: An overview. Eur. J. Med. Chem..

[2] Giannini G., Cabri W., Fattorusso C., Rodriquez M. (2012). Histone deacetylase inhibitors in the treatment of cancer: Overview and perspectives. Future Med. Chem..

[3] Suraweera A., O’Byrne K.J., Richard D.J. (2018). Combination Therapy With Histone Deacetylase Inhibitors (HDACi) for the Treatment of Cancer: Achieving the Full Therapeutic Potential of HDACi. Front. Oncol..

[4] Kong C.Y., Guo Z., Song P., Zhang X., Yuan Y.P., Teng T., Yan L., Tang Q.Z. (2022). Underlying the Mechanisms of Doxorubicin-Induced Acute Cardiotoxicity: Oxidative Stress and Cell Death. Int. J. Biol. Sci..

[5] Linders A.N., Dias I.B., Lopez Fernandez T., Tocchetti C.G., Bomer N., Van der Meer P. (2024). A review of the pathophysiological mechanisms of doxorubicin-induced cardiotoxicity and aging. npj Aging.

[6] Mattioli R., Ilari A., Colotti B., Mosca L., Fazi F., Colotti G. (2023). Doxorubicin and other anthracyclines in cancers: Activity, chemoresistance and its overcoming. Mol. Asp. Med..

[7] Hontecillas-Prieto L., Flores-Campos R., Silver A., de Álava E., Hajji N., García-Domínguez D.J. (2020). Synergistic Enhancement of Cancer Therapy Using HDAC Inhibitors: Opportunity for Clinical Trials. Front. Genet..

[8] Saha S.K., Yin Y., Kim K., Yang G.M., Dayem A.A., Choi H.Y., Cho S.G. (2017). Valproic Acid Induces Endocytosis-Mediated Doxorubicin Internalization and Shows Synergistic Cytotoxic Effects in Hepatocellular Carcinoma Cells. Int. J. Mol. Sci..

[9] Lai J.P., Sandhu D.S., Moser C.D., Cazanave S.C., Oseini A.M., Shire A.M., Shridhar V., Sanderson S.O., Roberts L.R. (2009). Additive effect of apicidin and doxorubicin in sulfatase 1 expressing hepatocellular carcinoma in vitro and in vivo. J. Hepatol..

[10] Hii L.-W., Chung F.F.-L., Soo J.S.-S., Tan B.S., Mai C.-W., Leong C.-O. (2020). Histone deacetylase (HDAC) inhibitors and doxorubicin combinations target both breast cancer stem cells and non-stem breast cancer cells simultaneously. Breast Cancer Res. Treat..

[11] Pang Y., Shi R., Chan L., Lu Y., Zhu D., Liu T., Yan M., Wang Y., Wang W. (2023). The combination of the HDAC1 inhibitor SAHA and doxorubicin has synergic efficacy in triple negative breast cancer in vivo. Pharmacol. Res..

[12] Lee S.J., Hwang S.O., Noh E.J., Kim D.U., Nam M., Kim J.H., Nam J.H., Hoe K.L. (2014). Transactivation of bad by vorinostat-induced acetylated p53 enhances doxorubicin-induced cytotoxicity in cervical cancer cells. Exp. Mol. Med..

[13] Olivieri M., Cho T., Álvarez-Quilón A., Li K., Schellenberg M.J., Zimmermann M., Hustedt N., Rossi S.E., Adam S., Melo H. (2020). A Genetic Map of the Response to DNA Damage in Human Cells. Cell.

[14] Abaza M.S., Bahman A.M., Al-Attiyah R.J. (2014). Valproic acid, an anti-epileptic drug and a histone deacetylase inhibitor, in combination with proteasome inhibitors exerts antiproliferative, pro-apoptotic and chemosensitizing effects in human colorectal cancer cells: Underlying molecular mechanisms. Int. J. Mol. Med..

[15] Catalano M.G., Fortunati N., Pugliese M., Poli R., Bosco O., Mastrocola R., Aragno M., Boccuzzi G. (2006). Valproic acid, a histone deacetylase inhibitor, enhances sensitivity to doxorubicin in anaplastic thyroid cancer cells. J. Endocrinol..

[16] Adimoolam S., Sirisawad M., Chen J., Thiemann P., Ford J.M., Buggy J.J. (2007). HDAC inhibitor PCI-24781 decreases RAD51 expression and inhibits homologous recombination. Proc. Natl. Acad. Sci. USA.

[17] Robert T., Vanoli F., Chiolo I., Shubassi G., Bernstein K.A., Rothstein R., Botrugno O.A., Parazzoli D., Oldani A., Minucci S. (2011). HDACs link the DNA damage response, processing of double-strand breaks and autophagy. Nature.

[18] Aleksandrova K.V., Vorobev M.L., Suvorova I.I. (2024). mTOR pathway occupies a central role in the emergence of latent cancer cells. Cell Death Dis..

[19] Liu Y., Azizian N.G., Sullivan D.K., Li Y. (2022). mTOR inhibition attenuates chemosensitivity through the induction of chemotherapy resistant persisters. Nat. Commun..

[20] Shibue T., Weinberg R.A. (2017). EMT, CSCs, and drug resistance: The mechanistic link and clinical implications. Nat. Rev. Clin. Oncol..

[21] Brochier C., Dennis G., Rivieccio M.A., McLaughlin K., Coppola G., Ratan R.R., Langley B. (2013). Specific acetylation of p53 by HDAC inhibition prevents DNA damage-induced apoptosis in neurons. J. Neurosci..

[22] Ito A., Kawaguchi Y., Lai C.H., Kovacs J.J., Higashimoto Y., Appella E., Yao T.P. (2002). MDM2-HDAC1-mediated deacetylation of p53 is required for its degradation. Embo J..

[23] Jenke R., Reßing N., Hansen F.K., Aigner A., Büch T. (2021). Anticancer Therapy with HDAC Inhibitors: Mechanism-Based Combination Strategies and Future Perspectives. Cancers.

[24] Sakaguchi K., Herrera J.E., Saito S., Miki T., Bustin M., Vassilev A., Anderson C.W., Appella E. (1998). DNA damage activates p53 through a phosphorylation-acetylation cascade. Genes. Dev..

[25] Tang Y., Zhao W., Chen Y., Zhao Y., Gu W. (2008). Acetylation is indispensable for p53 activation. Cell.

[26] Ellis L., Bots M., Lindemann R.K., Bolden J.E., Newbold A., Cluse L.A., Scott C.L., Strasser A., Atadja P., Lowe S.W. (2009). The histone deacetylase inhibitors LAQ824 and LBH589 do not require death receptor signaling or a functional apoptosome to mediate tumor cell death or therapeutic efficacy. Blood.

[27] Sonnemann J., Marx C., Becker S., Wittig S., Palani C.D., Krämer O.H., Beck J.F. (2014). p53-dependent and p53-independent anticancer effects of different histone deacetylase inhibitors. Br. J. Cancer.

[28] Verheijen M.C., Meier M.J., Asensio J.O., Gant T.W., Tong W., Yauk C.L., Caiment F. (2022). R-ODAF: Omics data analysis framework for regulatory application. Regul. Toxicol. Pharmacol..

[29] Li B., Dewey C.N. (2011). RSEM: Accurate transcript quantification from RNA-Seq data with or without a reference genome. BMC Bioinform..

[30] Love M.I., Huber W., Anders S. (2014). Moderated estimation of fold change and dispersion for RNA-seq data with DESeq2. Genome Biol..

[31] Ginestet C. (2011). ggplot2: Elegant Graphics for Data Analysis. J. R. Stat. Soc. Ser. A Stat. Soc..

[32] Engler J.B. (2025). Tidyplots empowers life scientists with easy code-based data visualization. Imeta.

