# Supplementary material for: HDAC Inhibitors Potentiate Doxorubicin Cytotoxicity in HepG2 Cells and Alter DNA-Repair- and p53-Associated Transcriptional Programs
Source: Int J Mol Sci. 2026 Oct 14;27(20):9091. doi: 10.3390/ijms27209091 (PMC13705291; doi:10.3390/ijms27209091)

Supplementary Figure S1

Uncropped image related to Figure 1A

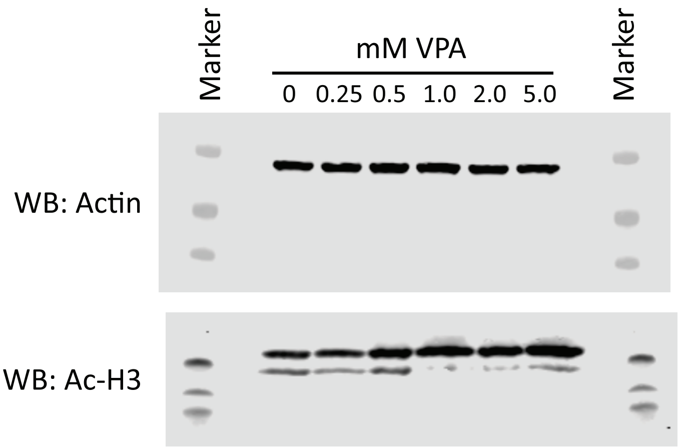

Second independent experiment

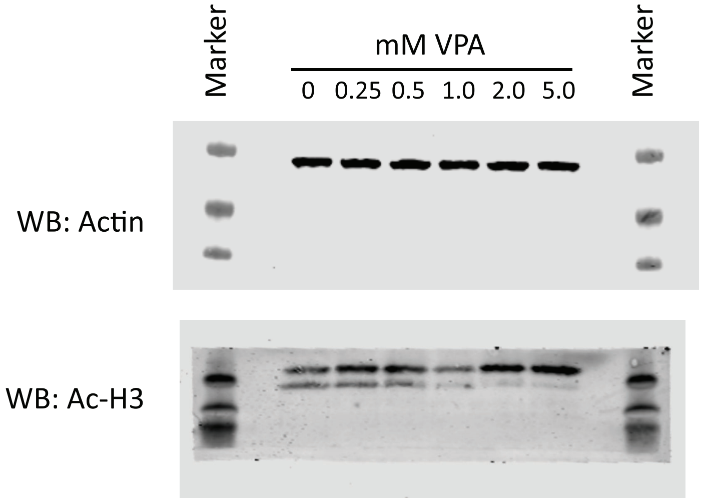

Uncropped image related to Figure 5C

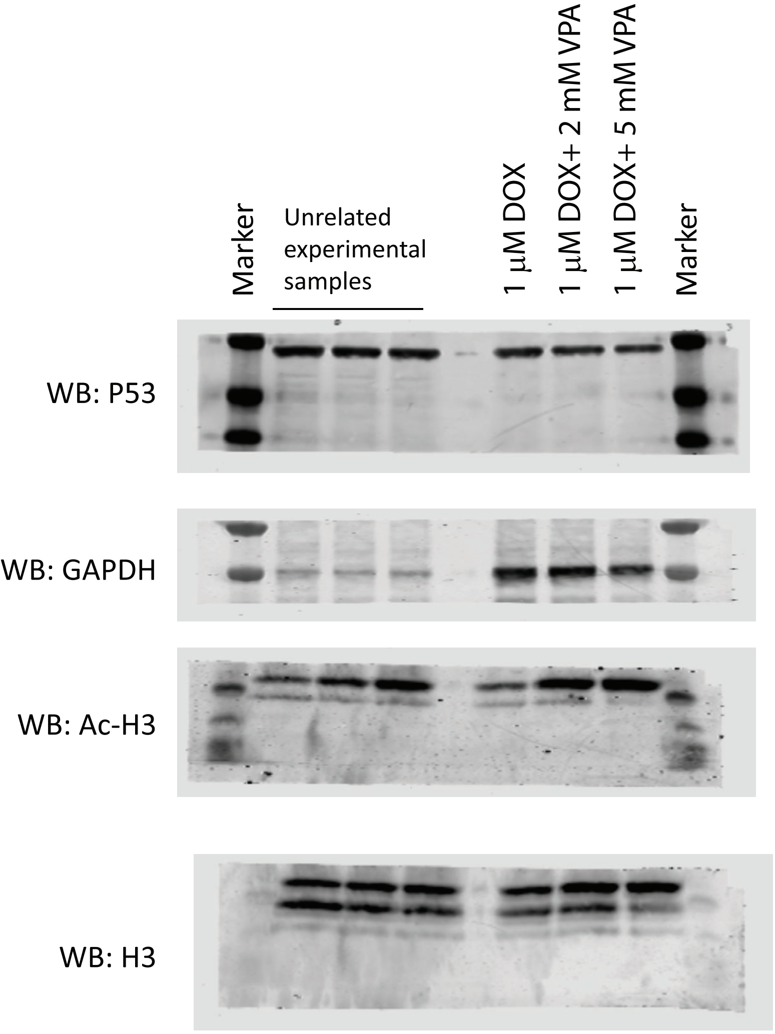

Uncropped image related to Figure 5D

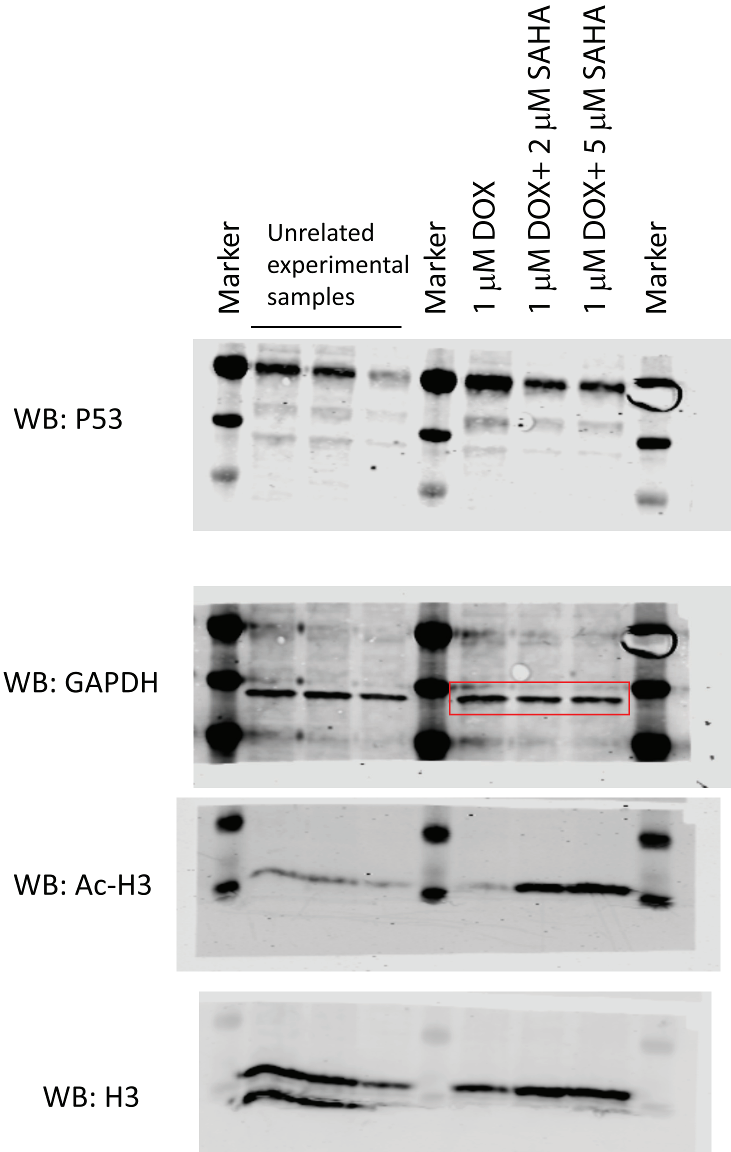

Supplement: Supplementary file 1 [file ijms-27-09091-s001.zip › Supplementary Figure S1.pdf]
