# Supplementary material for: HDAC Inhibitors Potentiate Doxorubicin Cytotoxicity in HepG2 Cells and Alter DNA-Repair- and p53-Associated Transcriptional Programs
Source: Int J Mol Sci. 2026 Oct 14;27(20):9091. doi: 10.3390/ijms27209091 (PMC13705291; doi:10.3390/ijms27209091)
Supplement: Supplementary file 1 [file ijms-27-09091-s001.zip › Supplementary FIgure S2.pdf]

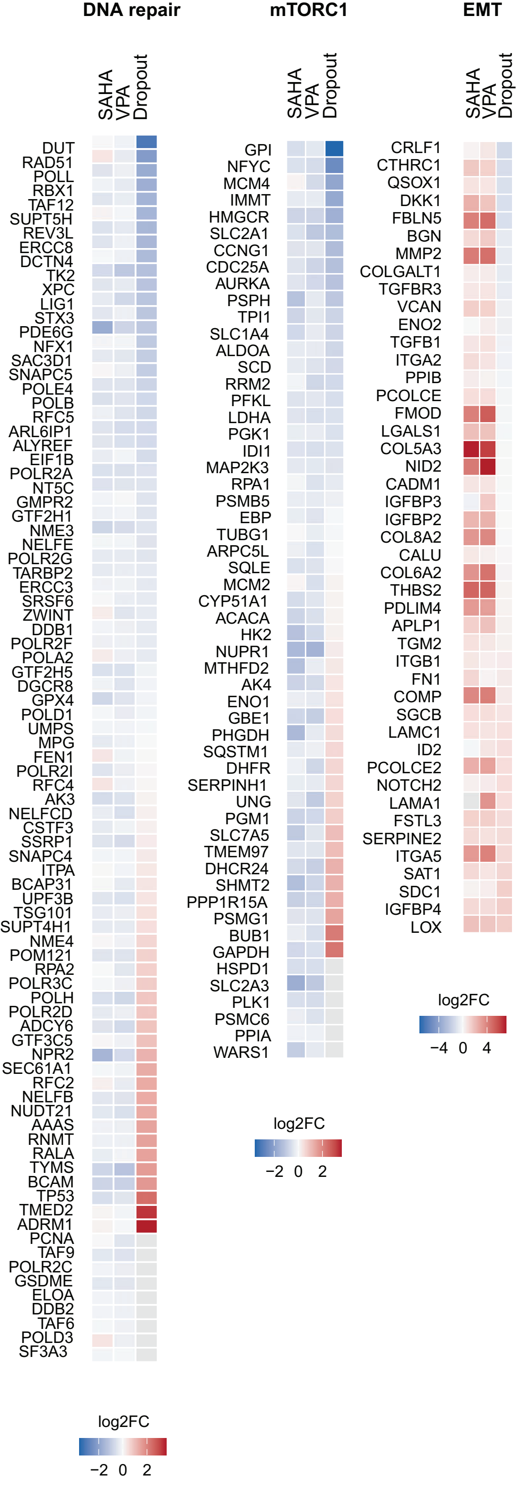
DNA repair
SAHA
VPA
Dropout
DUT
RAD51
POLL
RBX1
TAF12
SUPT5H
REV3L
ERCC8
DCTN4
TK2
XPC
LIG1
STX3
PDE6G
NFX1
SAC3D1
SNAPC5
POLE4
POLB
RFC5
ARL6IP1
ALYREF
EIF1B
POLR2A
NT5C
GMPR2
GTF2H1
NME3
NELFE
POLR2G
TARBP2
ERCC3
SRSF6
ZWINT
DDB1
POLR2F
POLA2
GTF2H5
DGCR8
GPX4
POLD1
UMPS
MPG
FEN1
POLR2I
RFC4
AK3
NELFCD
CSTF3
SSRP1
SNAPC4
ITPA
BCAP31
UPF3B
TSG101
SUPT4H1
NME4
POM121
RPA2
POLR3C
POLH
POLR2D
ADCY6
GTF3C5
NPR2
SEC61A1
RFC2
NELFB
NUDT21
AAAS
RNMT
RALA
TYMS
BCAM
TP53
TMED2
ADRM1
PCNA
TAF9
POLR2C
GSDME
ELOA
DDB2
TAF6
POLD3
SF3A3
log2FC
−2 0 2
mTORC1
SAHA
VPA
Dropout
GPI
NFYC
MCM4
IMMT
HMGCR
SLC2A1
CCNG1
CDC25A
AURKA
PSPH
TPI1
SLC1A4
ALDOA
SCD
RRM2
PFKL
LDHA
PGK1
IDI1
MAP2K3
RPA1
PSMB5
EBP
TUBG1
ARPC5L
SQLE
MCM2
CYP51A1
ACACA
HK2
NUPR1
MTHFD2
AK4
ENO1
GBE1
PHGDH
SQSTM1
DHFR
SERPINH1
UNG
PGM1
SLC7A5
TMEM97
DHCR24
SHMT2
PPP1R15A
PSMG1
BUB1
GAPDH
HSPD1
SLC2A3
PLK1
PSMC6
PPIA
WARS1
log2FC
−2 0 2
EMT
SAHA
VPA
Dropout
CRLF1
CTHRC1
QSOX1
DKK1
FBLN5
BGN
MMP2
COLGALT1
TGFBR3
VCAN
ENO2
TGFB1
ITGA2
PPIB
PCOLCE
FMOD
LGALS1
COL5A3
NID2
CADM1
IGFBP3
IGFBP2
COL8A2
CALU
COL6A2
THBS2
PDLIM4
APLP1
TGM2
ITGB1
FN1
COMP
SGCB
LAMC1
ID2
PCOLCE2
NOTCH2
LAMA1
FSTL3
SERPINE2
ITGA5
SAT1
SDC1
IGFBP4
LOX
log2FC
−4 0 4
