# Supplementary material for: HDAC Inhibitors Potentiate Doxorubicin Cytotoxicity in HepG2 Cells and Alter DNA-Repair- and p53-Associated Transcriptional Programs
Source: Int J Mol Sci. 2026 Oct 14;27(20):9091. doi: 10.3390/ijms27209091 (PMC13705291; doi:10.3390/ijms27209091)
Supplement: Supplementary file 1 [file ijms-27-09091-s001.zip › Supplementary figure S3.pdf]

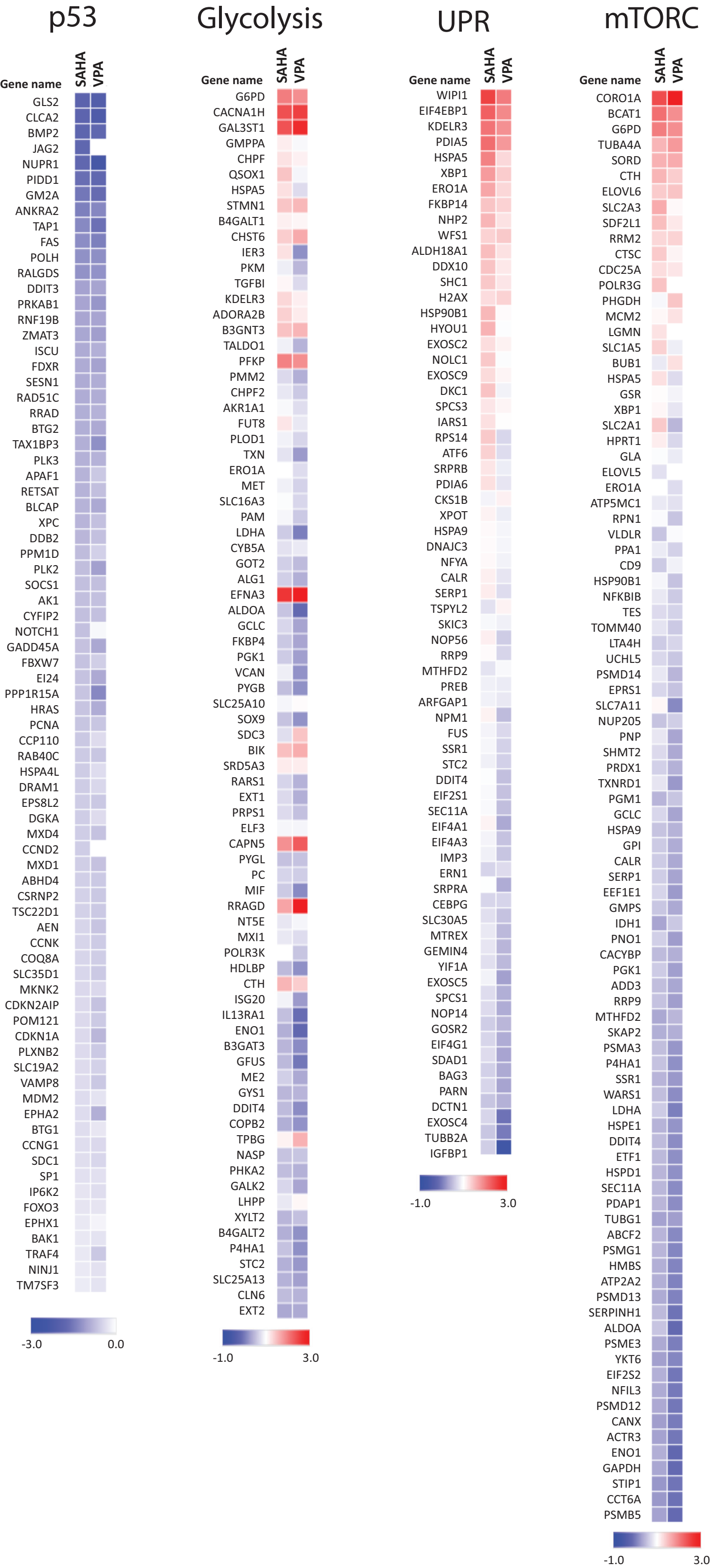
p53
Gene name
SAHA
VPA
GLS2
CLCA2
BMP2
JAG2
NUPR1
PIDD1
GM2A
ANKRA2
TAP1
FAS
POLH
RALGDS
DDIT3
PRKAB1
RNF19B
ZMAT3
ISCU
FDXR
SESN1
RAD51C
RRAD
BTG2
TAX1BP3
PLK3
APAF1
RETSAT
BLCAP
XPC
DDB2
PPM1D
PLK2
SOCS1
AK1
CYFIP2
NOTCH1
GADD45A
FBXW7
EI24
PPP1R15A
HRAS
PCNA
CCP110
RAB40C
HSPA4L
DRAM1
EPS8L2
DGKA
MXD4
CCND2
MXD1
ABHD4
CSRNP2
TSC22D1
AEN
CCNK
COQ8A
SLC35D1
MKNK2
CDKN2AIP
POM121
CDKN1A
PLXNB2
SLC19A2
VAMP8
MDM2
EPHA2
BTG1
CCNG1
SDC1
SP1
IP6K2
FOXO3
EPHX1
BAK1
TRAF4
NINJ1
TM7SF3
-3.0
0.0
Glycolysis
Gene name
SAHA
VPA
G6PD
CACNA1H
GAL3ST1
GMPPA
CHPF
QSOX1
HSPA5
STMN1
B4GALT1
CHST6
IER3
PKM
TGFBI
KDELR3
ADORA2B
B3GNT3
TALDO1
PFKP
PMM2
CHPF2
AKR1A1
FUT8
PLOD1
TXN
ERO1A
MET
SLC16A3
PAM
LDHA
CYB5A
GOT2
ALG1
EFNA3
ALDOA
GCLC
FKBP4
PGK1
VCAN
PYGB
SLC25A10
SOX9
SDC3
BIK
SRD5A3
RARS1
EXT1
PRPS1
ELF3
CAPN5
PYGL
PC
MIF
RRAGD
NT5E
MXI1
POLR3K
HDLBP
CTH
ISG20
IL13RA1
ENO1
B3GAT3
GFUS
ME2
GYS1
DDIT4
COPB2
TPBG
NASP
PHKA2
GALK2
LHPP
XYLT2
B4GALT2
P4HA1
STC2
SLC25A13
CLN6
EXT2
-1.0
3.0
UPR
Gene name
SAHA
VPA
WIPI1
EIF4EBP1
KDELR3
PDIA5
HSPA5
XBP1
ERO1A
FKBP14
NHP2
WFS1
ALDH18A1
DDX10
SHC1
H2AX
HSP90B1
HYOU1
EXOSC2
NOLC1
EXOSC9
DKC1
SPCS3
IARS1
RPS14
ATF6
SRPRB
PDIA6
CKS1B
XPOT
HSPA9
DNAJC3
NFYA
CALR
SERP1
TSPYL2
SKIC3
NOP56
RRP9
MTHFD2
PREB
ARFGAP1
NPM1
FUS
SSR1
STC2
DDIT4
EIF2S1
SEC11A
EIF4A1
EIF4A3
IMP3
ERN1
SRPRA
CEBPG
SLC30A5
MTREX
GEMIN4
YIF1A
EXOSC5
SPCS1
NOP14
GOSR2
EIF4G1
SDAD1
BAG3
PARN
DCTN1
EXOSC4
TUBB2A
IGFBP1
-1.0
3.0
mTORC
Gene name
SAHA
VPA
CORO1A
BCAT1
G6PD
TUBA4A
SORD
CTH
ELOVL6
SLC2A3
SDF2L1
RRM2
CTSC
CDC25A
POLR3G
PHGDH
MCM2
LGMN
SLC1A5
BUB1
HSPA5
GSR
XBP1
SLC2A1
HPRT1
GLA
ELOVL5
ERO1A
ATP5MC1
RPN1
VLDLR
PPA1
CD9
HSP90B1
NFKBIB
TES
TOMM40
LTA4H
UCHL5
PSMD14
EPRS1
SLC7A11
NUP205
PNP
SHMT2
PRDX1
TXNRD1
PGM1
GCLC
HSPA9
GPI
CALR
SERP1
EEF1E1
GMPS
IDH1
PNO1
CACYBP
PGK1
ADD3
RRP9
MTHFD2
SKAP2
PSMA3
P4HA1
SSR1
WARS1
LDHA
HSPE1
DDIT4
ETF1
HSPD1
SEC11A
PDAP1
TUBG1
ABCF2
PSMG1
HMBS
ATP2A2
PSMD13
SERPINH1
ALDOA
PSME3
YKT6
EIF2S2
NFIL3
PSMD12
CANX
ACTR3
ENO1
GAPDH
STIP1
CCT6A
PSMB5
-1.0
3.0
